# Aspirin: 120 years of innovation. A report from the 2017 Scientific Conference of the International Aspirin Foundation, 14 September 2017, Charité, Berlin

**DOI:** 10.3332/ecancer.2018.813

**Published:** 2018-02-20

**Authors:** Jaqui Walker, Pippa Hutchison, Junbo Ge, Dong Zhao, Yongjun Wang, Peter M Rothwell, J Michael Gaziano, Andrew Chan, John Burn, John Chia, Ruth Langley, Valerie O'Donnell, Bianca Rocca, Chris Hawkey

**Affiliations:** 1 International Aspirin Foundation, 34 Bower Mount Road, Maidstone, Kent ME16 8AU, UK; 2 Shanghai Institute of Cardiovascular Disease, 180 Fenglin Rd, Xuhui Qu, Shanghai Shi, China; 3 Center for Stem Cells and Tissue Engineering, Fudan University, 220 Handan Rd, WuJiaoChang, Yangpu Qu, Shanghai Shi, 200433, China; 4 Beijing Institute of Heart, Lung and Blood Vessel Diseases, Beijing, China; 5 Epidemiology Research Department, An Zhen Hospital, Beijing, China; 6 Beijing Tiantan Hospital, Capital Medical University, Beijing, China; 7 Centre for the Prevention of Stroke and Dementia, Nuffield Department of Clinical Neurosciences, University of Oxford, Oxford, UK; 8 Massachusetts Veterans Epidemiology Research and Information Center (MAVERIC), 150 South Huntington Avenue, Jamaica Plain, MA 02130, USA; 9 Harvard Medical School, 25 Shattuck St, Boston, MA 02115, USA; 10 Institute of Genetic Medicine, International Centre for Life, Times Square, Newcastle upon Tyne, NE1 3BZ, UK; 11 Department of Medical Oncology, National Cancer Centre, Bukit Merah, Singapore; 12 Brighton and Sussex University Hospitals, UK; 13 Medical Research Council Clinical Trials Unit, University College London, 90 High Holborn, London WC1V 6LJ, UK; 14 Systems Immunity Research Institute, Cardiff University, Tenovus Building, Cardiff, CF14 4XN, UK; 15 Catholic University School of Medicine, Rome, Italy; 16 Faculty of Medicine and Health Sciences, University of Nottingham, Medical School, Queen's Medical Centre, Nottingham, NG7 2UH, UK

**Keywords:** aspirin, inflammation, primary prevention, secondary prevention, tertiary prevention, colorectal cancer (CRC), cardiovascular disease (CVD), stroke, bleeding, risk, benefit, Lynch syndrome, precision medicine, individualised choice, personalised medicine

## Abstract

Acetylsalicylic acid was first synthesised by Dr FeIix Hoffman on 10th August 1897 and Aspirin was born. It quickly became the best-known pain killer in the world and in the 120 years since this event, aspirin has continued to attract interest, innovation and excitement. Set within the walls of the preserved ruins of Rudolf Virchow’s lecture hall at Charité, within Berlin’s Museum of Medical History, the International Aspirin Foundation’s 28th Scientific Conference served to facilitate international, multi-disease, multidisciplinary discussion about the current understanding of aspirin’s mechanisms of action and its utility in modern medicine as well as ideas for future research into its multifaceted applications to enhance global health.

In addition to the delegates in Berlin, 300 medical doctors at the 19th Annual Scientific Congress of the Chinese Society of Cardiology were able to join the cardiology sessions from Taiyuan, Shangxi province via a live streaming link to and from China. This led to useful discussion and allowed a truly international perspective to the meeting.

## First session—update on cardiovascular disease and stroke: East meets West

The first session, an update on cardiovascular disease (CVD) and stroke: East meets West was chaired by Professor Junbo Ge, who expressed his delight at joining the conference and working together to tackle CVD, which is now the number one killer in China.

### The disease burden of CVD and the major strategy of primary prevention for CVD in China—Dr Dong Zhao

Dr Dong Zhao joined the meeting via the live streaming link to the Chinese Society of Cardiology Congress in Taiyuan, and described the growing CVD burden in China and the primary prevention strategies used to tackle this. Dr Zhao is involved with the development of both the Chinese and the international CVD prevention guidelines.

CVD is currently the highest cause of premature death in China. In line with the United Nation’s sustainable development goals for 2030, China aims to reduce by a third all premature deaths from non-communicable causes [1]. In 2012, CVD was responsible for 41% of all urban deaths and 39% of rural deaths [2]. This challenge is heightened by the fact that the rate of mortality from ischaemic heart disease (IHD) has risen dramatically over the last 2 decades; it was the seventh leading cause of life lost in 1990, but by 2010 it had jumped up to be the second cause [3]. This pattern is expected to continue with the estimated numbers of people with IHD in China more than doubling from 8.1 million in 2010 to 22.6 million in 2030 [2–4]. Stroke statistics showed an improved survival rate and reduced disability [3, 5]; however, the number of people having a stroke is still expected to increase from 8.3 million in 2010 to 32 million in 2030. China ranks in the top three for premature deaths from stroke among the G20 countries [3], while ageing and population growth may account for at least half of the increase in CVD, lifestyle factors also play a key role in the current and future CVD epidemic.

Dr Zhao explained that CVD risk assessment is important in order to identify those at high-risk in the Chinese population without current CVD, and help them to understand the whole profile of their CVD risk factors in order to be able to provide an individualised CVD risk management plan. To be able to carry out CVD risk assessment, a CVD risk prediction model based on long-term observational studies and risk assessment tools are required as well as relevant recommendations in guidelines. Predictive models developed in one geographical area or ethnic population may not be suitable for other populations and regions. Risk assessment methods such as the Framingham CVD risk score, which has a homogeneous nature to its study population cannot be simply extrapolated to other settings. Specific tools of risk assessment for the Chinese population have been developed based upon the Chinese Multi-provincial cohort study.

The 2012 China National Plan for Non-Communicable Diseases Prevention and Treatment [6] has clear targets for CVD prevention. Both the 2016 China guidelines of dyslipidaemia management and the 2017 Chinese guidelines for CVD prevention (soon to be published) have new recommendations for risk assessment in China.

The 2016 China expert consensus advocates that for individuals with a 10-year arteriosclerotic cardiovascular disease [7] risk of greater than or equal to 10%, aspirin should be used for the primary prevention of CVD. Doctors in China need to identify those with a high CVD risk and provide early treatment for their CVD risk factors. The updated protocol of CVD risk assessment in China has provided a reasonable tool to assist clinicians in achieving this. Busy clinics in China can sometimes stand in the way of finding time to carry out CVD risk assessment; therefore, the greatest challenge will be finding ways to effectively implement these guidelines into CVD prevention practice in China.

### Antiplatelet therapy for stroke prevention in China—Professor Yongjun Wang

Professor Yongjun Wang from Beijing Tiantan Hospital presented from China on antiplatelet therapy for stroke prevention in China. Stroke is a major burden in China; from 2010, stroke became a leading cause of death and disability in China and mortality from stroke is five times higher in China than in the USA [3, 8]. According to a national population based survey in 2013, the prevalence of stroke in China was 1114.8 per 100, 000 people and its prevalence, incidence and mortality rates were significantly higher in rural areas compared with urban areas [9]. More than 70% of stroke patients have either ischaemic stroke or transient ischaemic attack (TIA) [9].

The Chinese clinical guidelines for the secondary prevention of ischaemic stroke and TIA recommend an optimal dosage of aspirin between 75 mg/day and 150 mg/day. A combination of aspirin and clopidogrel for 21 days is recommended for patients with minor stroke or high-risk TIA within 24 hour of onset [10]. These recommendations are based on the CHANCE trial [11].

The rationale behind the randomised controlled CHANCE trial was to find the ‘sweet spot’ or balance point of efficacy and safety for dual antiplatelet therapy in stroke. After three previous trials, MATCH 2004, PRoFESS 2008 and SPS3 2012, all failed to increase efficacy but increased the risk of bleeding, CHANCE, which randomised 5,170 patients from 114 hospitals focused on early, short-term, optimised dual antiplatelet therapy (within 24 hours) to reduce the risk of new stroke at 3 months for clopidogrel-aspirin treatment in acute minor stroke or high-risk TIA. After a big data analysis based on 90,000 patients in other trials, several strategies were found that may improve the efficacy, without increasing the risk of bleeding, of antiplatelet therapy. This formed the rationale behind CHANCE;

1. Intensive antiplatelet therapy should be initiated within 24 hours.
2. High-risk non-disabling patients may have high risk of ischaemic events but low risk of bleeding and are therefore the appropriate target population.
3. An appropriate treatment strategy with a loading dose of clopidogrel 300 g given as soon as possible and then dual antiplatelet for 21 days should be used.

The hypothesis was that this early, short-term, optimised dual antiplatelet therapy could be an effective strategy with a low risk of bleeding [12]. CHANCE showed an early benefit of clopidogrel-aspirin treatment in reducing the risk of subsequent stroke, which persisted for the duration of the 1-year of follow-up [11]. They also found in patients treated within 12 hours, the combination of clopidogrel and aspirin was more effective than taking aspirin alone in reducing the risk of recurrent ischaemic stroke during the 90-day follow-up and did not increase the haemorrhagic risk [13]. Clopidogrel-aspirin treatment may have a benefit of reducing stroke risk outweighing the potential risk of increased bleeding especially within the first 2 weeks compared with aspirin alone in patients with minor stroke or TIA [14]. In addition, two-week combination therapy may be enough for minor stroke or high-risk TIA.

The CYP2C19 genotype and clopidogrel responsiveness were also investigated in the CHANCE trial, and it was discovered that the use of clopidogrel plus aspirin compared with the use of aspirin alone reduced the risk of a new stroke only in the subgroup of patients who were not carriers of the CYP2C19 loss-of-function alleles. In CHANCE, it was found that nearly 59% of patients were carriers of the CYP2C19 loss-of-function alleles [15]. These findings support a role of the CYP2C19 genotype in the efficacy of this treatment. Individualised antiplatelet therapy according to genotype will help target treatment in the future.

However, the efficacy of ticagrelor is not affected by CYP2C19 and this led to the development of another randomised controlled trial (RCT), the PRINCE trial, in order to assess 90-day platelet reactivity for ticagrelor-aspirin treatment compared with clopidogrel-aspirin treatment in acute minor stroke or high-risk TIA within 24 hours after onset. The interim analysis suggests that looking at platelet reactivity units and high on-treatment platelet reactivity; there may be a positive trend for better efficacy for those treated with the ticagrelor and aspirin combination.

Professor Wang concluded that antiplatelet therapy is easy to use, inexpensive and well tolerated; however, adherence is not optimal with a decline in antiplatelet use from 81% at the time of hospital discharge to 66% post-stroke [16].

### Acute effects of aspirin in TIA and stroke—Professor Peter Rothwell

Professor Peter Rothwell, Head of the Centre for the Prevention of Stroke and Dementia in Oxford, UK took the delegates through the role of aspirin in acute TIA and minor stroke. There is an acute or early risk of a major stroke occurring after TIA or minor stroke of around 10%, unless appropriate treatment is given [17–20]. The first hours after a minor stroke or TIA are therefore considered an emergency. Unfortunately many patients after a TIA or minor stroke wait more than 24 hours to see their GP; the length of the delay depends on the day of the week, with weekends leading to a longer wait [21].

Despite UK public education such as the FAST campaign (Face, Arms Speech, Time https://www.stroke.org.uk/take-action/recognise-signs-stroke), people are still not seeking medical attention soon enough [22]. Unlike a suspected heart attack, where the American Heart Association and British Heart Foundation advise self-medication with an aspirin, official website advice for a suspected TIA or stroke varies with either no recommendation or advice to check with a doctor before taking aspirin. Pre-hospital self-administration of aspirin tends to be discouraged after stroke because of concerns about the possible risk of intracerebral haemorrhage. This fear is unfounded as haemorrhage is a rare cause of TIA symptoms and is responsible for less than 5% of minor strokes. People should be encouraged to seek immediate medical attention, and with transient neurological symptoms, self-administration of aspirin may also be appropriate especially where access to medical care may be delayed.

There are few randomised trials on the effect of aspirin on the risk of early recurrent stroke after TIA and minor stroke and no data on its severity. However, observational studies suggest early substantial benefits.

The EXPRESS study which was not randomised but a before and after study showed an 80% reduction of recurrent stroke when patients were seen promptly and given aspirin, started on a statins and BP lowering agents [23, 24]. Of these, aspirin is hypothesised to produce much of the acute benefit [23]. The severity of recurrent cerebral events was also reduced in EXPRESS.

Due to the absence of published randomised evidence, data were extracted and re-analysed from all available previous trials of aspirin versus placebo for secondary prevention after TIA or ischaemic stroke [25]. This showed that the acute benefits of aspirin have been underestimated. The researchers found that if aspirin is given early after TIA and minor stroke, there are less severe recurrent events as well as far fewer events.

Professor Rothwell ended his presentation recommending that the general public should self-administer aspirin after TIA in the same way that they take aspirin for chest pain. Timely medical treatment with aspirin as a key intervention is important after all possible TIAs or minor stroke. Aspirin should be self-medicated after an unknown ‘funny turn’, it is widely available, costs virtually nothing and there is no major bleeding issue after transient events.

### Primary prevention in the US and Europe and forthcoming trials—Professor Mike Gaziano

At the other end of the spectrum Professor Gaziano, a preventive cardiologist and internationally recognised chronic disease epidemiologist from Boston, USA, spoke of his work in aspirin primary prevention trials including the landmark Physicians Health Study, the large-scale Women’s Health Study and currently, the ARRIVE trial.

Whilst the benefit of aspirin antiplatelet therapy has been clearly demonstrated for people with previous CVD [26], this risk/benefit equation is more complex in primary prevention; where individuals are at a risk of an initial CVD but have not yet had an event.

The United States Preventative Services Task Force (USPSTF) [27] has conducted a systematic review of the effect of aspirin in the primary prevention of CVD. It is worth noting that the primary prevention trials were mostly among people of European descent, and the risk-to-benefit ratio may differ in other populations. USPSTF used a series of scholarly works to underpin their decisions about aspirin in the primary prevention of CVD and colorectal cancer (CRC). They looked at the number of events prevented versus the number of events caused (e.g., gastrointestinal (GI) bleeding) and considered absolute number of events, all risk in net life years and quality of life years gained. This was a multiple risk strategy. Following their review, the USPSTF has recommended initiating low-dose aspirin use for the primary prevention of CVD and CRC in adults aged 50–59 years who have a 10% or greater 10-year CVD risk, who are not at risk of bleeding, have a life expectancy of at least 10 years, and are willing to take low-dose aspirin for at least 10 years. They also stated that in the 60–69 year age group, the decision to initiate aspirin should be an individual one and that the evidence available is insufficient to recommend for or against the use of aspirin in the prevention of CVD and CRC in those, who are younger than 50 years and 70 years or above.

It is the action of aspirin on platelet function that works to prevent CVD events that is also responsible for its side effect of bleeding risk. This bleeding risk falls into two main categories: GI bleeding and intracranial bleeding. Risk of bleeding can increase with a person’s age, gender, medication use and CVD risk factors [28].

While numerous trials have been carried out on the role of aspirin in acute treatment and the secondary prevention of CVD, there are relatively few in the area of primary prevention and the most have been done among those at lower risk of CVD events. This is because of the large scale that is required for these studies and the need for long-term follow-up. However, several on-going primary prevention trials are now under way and will come to fruition over the next few years, which will help to give more insight into the use of aspirin in populations where the risk of CVD is higher than the general population. ARRIVE (Aspirin to Reduce Risk of Initial Vascular Events) tests aspirin 100 mg daily in those with moderate-to-high risk of a CVD event results are expected in 2018. ARRIVE is being conducted mainly in Europe. ASPREE (Aspirin in Reducing Events in the Elderly) looks at patients over 70 years and is being conducted in Australia and the United States; ASCEND is a study based in the UK, testing low-dose aspirin in diabetics without known CVD and ACCEPT-D tests low-dose aspirin and simvastatin in diabetics. These trials will provide critical information to refine recommendations for different segments of the population.

People should be encouraged to talk with their clinician about starting low-dose aspirin. Providers also need education on how to apply risk assessment tools and make calculations regarding the use of low-dose aspirin. In this age of empowerment, patients can help with this dialogue. Tools are emerging that help patients own more of their health data and contribute to decisions about their treatment. An assessment of CVD risk, total mortality, cancer risk and bleeding risks are necessary, when considering aspirin in primary prevention. Therefore, unlike acute care, where a more parental role is used to recommend treatment, in primary prevention, we need to empower patients to make the decision. In an acute event, the patient can see the benefit of the therapy and understands the risk; but in primary prevention, the patient often feels well and yet their blood pressure and cholesterol are raised. They might need to take a statin, aspirin and blood pressure medication. All of which requires active participation from the patient. ‘The person won’t thank you for the heart attack or stroke they didn’t have but they may blame you for a bleed while on aspirin’. This is a difficult paradigm and a complex construct for the clinician to manage.

Professor Gaziano called for a consideration for wider use of low-dose aspirin in both primary and secondary prevention, which could potentially save hundreds of thousands of lives annually and prevent millions of CVD and cancer events around the world.

During the discussion time, the question of what is low-dose aspirin in primary prevention was raised. Overall there is a lack of data to agree an exact dose but when using aspirin for primary prevention compliance over a long time period needs to be considered, and it is therefore better to be within an effective dose range rather than on the margin of the dose range; 100 mg a day seems to be effective for most people. However, a one dose fits all approach may not be right. There is currently no tailoring to weight and physical characteristics and this is probably what is needed especially where compliance is an issue leading to an intermittent rather than continuous antithrombotic effect. Personalised medicine will probably be the future with precise targeting of medication. This is an area of active research currently with a large trial in secondary prevention testing two doses head to head.

Stopping aspirin for surgery was also discussed. In many instances this is performed more for the the surgeons’ benefit as the operation will be approximately 10% shorter but is not usually necessary for the patient. Surgery places the patient into a very prothrombotic state, so stopping aspirin is not helpful.

Delegates asked how long patients should be on aspirin. It was felt that overall in primary care in general there should be on-going dialogue regarding CVD risk, cancer risk, life expectancy and bleeding risks on aspirin, as these all change over time. Therefore, at fairly regular intervals, medications need to be reviewed with the patient in order to update where the patient is on the risk to benefit spectrum.

## Oncology session

The oncology session, chaired by Professor Ruth Langley (Brighton and Sussex University Hospital and University College London, UK), looked at the evidence for aspirin in cancer prevention and treatment and the challenges in communicating the benefits and risks of aspirin to the wider population.

### Aspirin for the primary prevention of colorectal cancer—Professor Andrew Chan

Professor Andrew Chan, who heads the Clinical and Translational Epidemiology Unit at Massachusetts General Hospital and is a professor of medicine at Harvard Medical School, spoke about the role of aspirin for the primary prevention of CRC. He explained that in some ways this should be a public health consideration in the context of aspirin’s role in multiple diseases rather than any single individual disease. An improved identification of those in whom the protective effect of aspirin outweighs any harms will be important, and this precision medicine approach may emerge as further understanding of aspirin’s mechanism of action become clearer.

Colon cancer is one of the most preventable cancers in the developed world; however, the current methods of prevention do have limitations [29]. There is however a substantial weight of evidence supporting aspirin in CRC prevention starting with research carried out in the 1980s and continuing through to the current day. The data include case control and cohort studies as well as some RCTs. Current cancer screening methods are successful but there are limitations and they are resource intensive limiting its potential in many populations. To investigate the potential for precision medicine, Professor Chan’s team leveraged data from two large population-based cohorts. From a pooled-analysis of ten cohort and case-control studies, aspirin was shown to reduce the risk of CRC by 29% [30]. Further data have come from a secondary analysis of RCTs; a meta-analysis of four RCTs suggested that aspirin treatment for five or more years at doses of at least 75 mg daily reduced long-term CRC risk by 24% [31]. It appeared that a significant reduction in risk was not observed until at least a decade of use. However, aspirin use is associated with clear hazards – especially GI bleeding and therefore, there is a critical need for a precision medicine approach.

The weight of the evidence is so strong that the influential USPSTF released a recommendation which incorporates CRC prevention into the rationale for recommending routine aspirin use among patients with cardiovascular risk factors. Although this guideline doesn’t speak to a recommendation in the absence of CVD prevention, it is a milestone in that, other than tamoxifen in high risk breast patients, this is the first medication recommended for cancer prevention.

Professor Chan explained that a definitive RCT testing long-term daily aspirin at a range of doses for its effect on CRC is difficult to carry out due to the large number of subjects and the length of follow-up that would be required. Instead RCTs designed to test the effect of aspirin on colorectal adenomas, the precursor to most CRCs, have been carried out and have provided positive data for aspirin in chemoprevention. These include the Aspirin/Folate Polyp Prevention Study, Association pour la Prévention parl’Aspirine du Cancer Colorectal, Cancer and Leukemia Group B, the United Kingdom Colorectal Adenoma Prevention trial and the Japan Colorectal Aspirin Polyps Prevention trial.

In future years, additional RCTs such as ARRIVE, ASCOLT, ASPIRED, ASPREE, CAPP3, SeAFOod and the continued collection of long-term outcome data from completed trials are expected to add to the growing weight of evidence supporting the use of aspirin for the prevention of CRC.

Professor Chan proposed that aspirin probably works via an integrative multi-pathway model rather than any single dominant pathway for its mode of action. As our understanding of aspirin’s anti-cancer mechanism grows, it may be possible to develop molecular biomarkers (using tissue, urinary or genetic biomarkers) that will help to stratify those most likely to benefit from taking long-term aspirin. Pathways for finding potential biomarkers include the inhibition of prostaglandin-endoperoxide synthase, the Wnt/β-catenin signalling axis, aspirin’s anti-inflammatory properties (including host immune response modulation) and platelet mediated effects.

As well as these effects on cancer initiation, aspirin may also inhibit cancer progression. A number of studies have shown aspirin use to lower cancer specific CRC mortality in patients with CRC [32–38]. This action may be explained by aspirin’s effect on prostaglandins (PG) synthesis or Wnt signalling or other mechanisms such as the PIK3CA mutant. Biomarkers such as the PIK3CA mutation may be used in the future, in order to predict responsiveness to aspirin treatment.

Professor Chan concluded that there is overwhelming evidence to support the chemo preventative benefit of aspirin on CRC. Hazards associated with long-term aspirin use, such as GI bleeding, make strategies for risk stratification important and work around aspirin’s mechanisms of action may help to target its application to specific groups. Aspirin has an integrative multi-pathway model for its mode of action, and it may be possible to use these pathways to develop mechanistic biomarkers for personalised risk stratification. Molecular and generic markers in prostaglandin and inflammatory pathways hold promise. Such biomarkers could then be translated clinically to predict, who will benefit from aspirin chemoprevention and treatment and fulfil the promise of a precision medicine approach.

### Lynch syndrome and experience of implementing secondary prevention—Professor Sir John Burn

Lynch syndrome is an important area of investigation for aspirin and its cancer preventative effects because it provides a genetic subgroup of people, who can be specifically treated with the aim of benefiting them and informing the research into aspirin’s mechanism in cancer prevention as a whole.

Professor Sir John Burn (Newcastle University, UK) presented an analysis of cancer rates carried out at the 10th year of the CAPP2 trial, which showed that aspirin has a protective effect against cancer [39]. A secondary analysis of the impact of obesity revealed that individuals, who had the genetic predisposition for cancer and were overweight were more than twice as likely to develop CRC. This effect was partially abrogated in the aspirin arm of the study when compared with placebo possibly due to the anti-inflammatory role of the 600 mg dose used in the study. The 10-year blinded follow-up of this study is expected to confirm that there is a protective effect from aspirin use and that this effect is a truly preventative effect rather than suppressing tumours, which may then later emerge. In most cases, the aspirin was discontinued in CAPP2 before the impact on cancer incidence. This suggests an impact on precancerous legions (possibly by enhanced apoptosis or immune clearance of defective stem cells) rather than a direct effect on malignant cells.

CAPP3, a randomised dose non-inferiority trial will compare the 600 mg dose of aspirin, shown to be effective in CAPP2, with a 300 mg and 100 mg daily dosing schedule. Results should be available from 2023 with an adverse events assessment in 2020. Additionally, a biobank will assess frame shift peptide antibody titres a possible biomarker of subclinical cancer development.

### Aspirin for cancer prevention and cure—Is the time now? Professor John Chia

Professor Chia (National Cancer Centre, Singapore) described the twin challenges of CVD and cancer facing Asia with its current burgeoning populations. Over the upcoming decades, the economic burden of these two major diseases is expected to cost in excess of a trillion dollars [40].

Professor Chia reviewed past and on-going trials of aspirin in cancer and asked if the widespread adoption of aspirin as a chemo preventative agent in Asia is an idea whose ‘time has come’.

As well as a primary prevention and treatment role for CRC, aspirin is also creating interest for its role in tertiary cancer prevention via its synergy with immunotherapy. The 2017 European Society for Medical Oncology (ESMO) conference was dominated by immune oncology and headline press currently are the immune check point inhibitors such as pembrolizumab and nivolumab. Immunotherapy is set to become the fourth pillar of cancer therapy along with surgery, radiotherapy and chemotherapy.

Aspirin now also has a story to tell in combination with these new compounds, as it appears to have a synergistic effect with them. Aspirin in combination with immunotherapy is of key interest and it is being included in some immune check point inhibitor trials. Aspirin also potentially enhances adoptive T cell therapy and this unfolding story is thought to be due to the fact that platelets potentially stop T cells from killing cancer cells. Aspirin appears to allow the immune system access to kill cancer cells. These examples of aspirin’s exciting and potentially synergistic role in cancer immune therapy created a lot of discussion among the delegates at the meeting.

### Discussion about clinical implications—Professor Ruth Langley

The discussion focused on how best to communicate aspirin’s benefits and risks, and its role in cancer prevention both primary and secondary as well as tertiary/adjuvant treatment of micro metastatic disease [41]. The concept of chemoprevention for cancer is relatively novel and particularly in primary prevention, is a fairly complex concept to grasp particularly as the effects of aspirin take about 10 years to become apparent and have to be balanced with the risks of potential toxicity, particularly serious bleeding. The challenge of explaining the risks and benefits to patients was discussed, and it was felt that the development of a measurable biomarker analogous to cholesterol or blood pressure in CVD may be required for therapeutic cancer prevention to be successful and implemented.

The group agreed that these complicated messages with many factors to communicate represented a major challenge. Social media with its short punchy lines may not be appropriate. Instead it was felt that primary care clinicians could be an important target for education and information about the risks and benefits of aspirin. However, others felt that in the modern 21st century, we need to move away from a professional telling you what to do and that people need to make their own informed decision. The evidence is there, it has been 30 years in making, and individuals can decide to reduce their CVD and cancer risk in exchange for a small risk of side effects.

Other issues considered included the fact that aspirin is not considered to be an oncology drug, and meetings and conferences are arranged by tumour site which may be a challenge for chemoprevention. Understanding the anti-cancer mechanism(s) of action of aspirin will be crucial to maximising its clinical utility in the future.

## Science session

The science session chaired by Professor Lina Badimon (Cardiovascular Research Center, Spain) explored some of the excellent work currently being carried out to enhance our understanding of aspirin’s mode of action and pharmacological profile.

### The aspirin-sensitive platelet lipidome: beyond thromboxane A2 (TXA2)—Professor Valerie O’Donnell

Valerie O’Donnell is a Professor of biochemistry and Co-Director of the Systems Immunity Research Institute in Cardiff. Professor O’Donnell uses new generation mass spectrometry to characterise cellular lipidomes. Aspirin’s cardioprotective effects result from it blocking cyclooxygenase-1 dependent generation of the pro-thrombotic lipid TXA2. In her work Professor O’Donnell uses high resolution mass spectrometry techniques in order to gain insight into the behaviour of cellular lipidomes in health and disease. By defining the aspirin sensitive platelet lipidome and investigating how platelet lipids change with aspirin treatment, we can further enhance the diagnosis and treatment of patients. In particular Professor O’Donnell has set out to gain answers to the following questions:

- How many types of lipids do platelets contain?
- Can we use this information to discover new bioactive lipids from platelets?
- How do lipids vary over time in the same people?
- What is the effect of gender on platelet lipids?
- How variable are aspirin responses in the same people over time?

Understanding the total diversity and number of individual lipids in cells as well as how they alter during activation of cells and differ between individuals will help to improve the understanding of lipid biochemistry, and helps to find new targets for drug therapy and improve the identification of lipid biomarkers in cohort samples. In particularly this work will help to identify bioactive lipids that are usually present in very small amounts and therefore, not routinely detected.

Following some innovative research work using mass spectrometry, informatics, statistics and the development of some in-house software to manage data, Professor O’Donnell and her team began to characterise the platelet aspirin-sensitive lipidome and use this to uncover lipidomic networks [42]. They found that the human platelet lipidome is complex and that major changes occurred following the ingestion of aspirin. They found large numbers of lipids appeared on activation and of these in excess of 70% were sensitive to aspirin. There is a lot of potential discovery science in finding new lipids and this represents an important opportunity for the future.

After an initial pilot in three genetically different donors, a current project is underway looking at 30 volunteers over a six month period during which they were given repeated aspirin. The results of this work are expected soon. The research is still at the stage of trying to understand what goes on in healthy people rather than exploring effects in disease.

### Pharmacokinetic/pharmacodynamic (PK/PD) determinants of the inter-individual variability in the antiplatelet response: aspirin ‘resistance’ revisited—Professor Bianca Rocca

Professor Bianca Rocca from the pharmacology Institute of the Catholic University School of Medicine in Rome discussed the PK and PD determinants of the interindividual variability in the antiplatelet response and aspirin ‘resistance’.

She explained that many variables contribute to the clinical outcome, starting from the prescribed dose of each drug: the prescribed dose of a drug may differ from that actually administered, the dose/drug concentration at the site of action and the intensity of pharmacological effect can be affected by physiological (e.g., age, renal function etc.), pathological factors (e.g., kidney or liver dysfunctions), genetic variation, drug-drug interactions and the development of resistance or tolerance.

Understanding the determinants of inter-individual variability in drug responsiveness and reducing it can improve a drug’s effectiveness in real world settings. If reliable biomarkers can be found, a greater understanding of determinants of drug variability can be developed, and this will inform the development of the personalised, precision or stratified medicine. Serum TXB2 ex vivo is for example a PD biomarker supported by the European Medicines Agency, which can be used to check the efficacy of new aspirin formulations [43].

Inter-individual variability in response applies to aspirin as well, and seems to be associated with its PD and/or metabolic disposition, using the correct assay appears crucial for identifying determinants of variability and designing new regimens. Identifying determinants of variability in aspirin response is relevant in hypothesising and testing new ways of administering an ‘old’, effective (and cheap) drug in selected clinical conditions.

Shortening the dosing interval rescues the impaired antiplatelet effect of low-dose aspirin in acute or chronic settings of high platelet turnover. A reduced bioavailability may benefit from doubling the once-daily dose. The clinical translation of these PD/PK findings will need adequately sized randomised trials comparing improved versus conventional regimens.

Essential thrombocythaemia (ET) (a myeloproliferative neoplasm, which causes increased platelet generation and an increased risk of thrombotic complications) is one example, where the dose of aspirin may need to be altered and algorithms for treating ET at intermediate and high risk of thrombosis have been developed with twice-daily aspirin being considered for certain patients [44].

Another area, where aspirin dosing may require temporary variation is in the acute setting soon after cardiac surgery for CABG, unless the bleeding risk is high, work has now shown that a twice daily low-dose aspirin regimen may be required, especially in the first 3 months post-surgery [45].

The global obesity epidemic is another issue for drug PK with a multitude of changes that take place and a speeding up metabolism and excretion. The net effect of which on a single drug is variable and unpredictable especially for lipophilic drugs. Aspirin is lipophilic and body weight has been shown to reduce aspirin efficacy in inhibiting platelet thromboxane [46]. The area of obesity and drug PK is currently a big gap in knowledge especially in patients with CVD and with a body mass index (BMI) above 35 or 40 kg/m^2^.

Inter-individual variability in response to aspirin is an important issue and is associated with its PD and PK. Research into this area of pharmacology using the correct assays will be crucial, in order to identify the determinants of this variability and design new regimens.

### What is the risk of bleeding?—Professor Peter Rothwell

Professor Rothwell explored the risk of bleeding in the secondary prevention setting, where lifelong antiplatelet treatment is recommended after a vascular event. Antiplatelet drugs increase the risk of a patient experiencing a major bleed in particular upper- GI bleeding [47]. These potential harms include haemorrhagic stroke and GI bleeding. The risk of haemorrhagic stroke is largely offset by the reductions in ischaemic stroke and the HOT trial showed that optimising blood pressure control can help to minimise the haemorrhagic strokes caused by aspirin. In the case of GI bleeding, the majority of people do not have serious sequelae with this if they are under the age of 65 years.

Proton pump inhibitors (PPI) do however reduce the risk of a GI bleed by 70–90% [48] but co-prescription is not currently routine practice. Secondary prevention clinical guidelines make no recommendations on PPI use and although some consensus statements do advocate GI protection in ‘high risk’ patients, the definition of who is at high risk varies. The low uptake of PPI use may be due to concerns about long-term harm from PPIs and variability in the definition of who constitutes the ‘high risk’ population for bleeding on aspirin. The evidence behind antiplatelet therapy in secondary prevention is based on early randomised trials in patients below 75 years. However, the mean age for a myocardial infarction (MI) is 75 years with the mean age for TIA and stroke is 65 years. It is estimated that 50% of patients taking antiplatelet are now at the age of 75 years and older.

The Oxford Vascular Study (OXVASC) is a prospective, population based study of all incident and recurrent acute vascular events including TIA, ischaemic stroke and MI. It is based on a population of 92,728 people. In OXVASC, 3166 patients of which 1094 had an MI and 2072 had a TIA/ischaemic stroke were studied and all were treated with antiplatelet medication. At one year, 2,301 (89%) were on antiplatelet treatment and 852 (33%) were taking a PPI or H2 antagonist. Half (1,582 (50%)) of the patients were 75 years or older. The study found that during 13,509 years of patient follow-up there were 405 first bleeding events and of these 162 were upper GI [49]. The risk of having a major bleed increased steeply with age and was more severe and more sustained at older ages. The study group found the estimated number needed to treat (NNT) for routine PPI use to prevent one disabling/fatal upper-GI bleed over 5 years fell from 338 at ages less than 65 years to 25 at aged 85 years or older.

Professor Rothwell concluded that the long-term risks and severity of bleeding on aspirin-based antiplatelet treatment in secondary prevention increase steeply with age [49]. For those under 75 years, the risks are comparable with those reported in RCTs. In patients under 75 years, the bleeding risk is more front loaded to the first year and flattens after this time period. In those 75 years or older, the risks of bleeding are higher, more severe and more sustained and the functional outcomes are far worse. In patients 75 years or older, upper GI bleeds tend to be major disabling or fatal bleeding events. Professor Rothwell recommended the routine use of PPIs in those 75 years or older to prevent upper GI bleeds when taking aspirin. He suggested that this be considered for inclusion in future secondary prevention guidelines.

### Causes of bleeding and strategies for prevention—Professor Chris Hawkey

Professor Hawkey (University of Nottingham, UK) explained that in contrast to non-steroidal anti-inflammatory drugs (NSAIDS) there is a clear clinical and epidemiological evidence that patients with the bacteria Helicobacter Pylori (H.Pylori), who are also taking aspirin have an increased risk of ulcer development and bleeding [50]. This may be due to the fact that one of the main actions of aspirin is to abrogate haemostasis and promote bleeding in lesions caused by another agent, e.g., H. pylori, while with NSAIDS it is their intrinsic ulcerogenic activity, which is the important factor.

Therefore two main strategies are proposed to protect against aspirin associated ulceration and ulcer bleeding;

- Use of an ulcer healing agent
- H. pylori eradication

Ulcer healing agent: in a meta-analysis of ten RCTs involving 8,780 participants, PPIs were found to be superior to both H2 receptor antagonists and gefarnate in preventing ulceration or bleeding [48]. There are concerns about the risk of long-term use of PPIs [51]. It is interesting therefore that a recent trial has found famotidine (H2 receptor antagonist) to have equal efficacy to a PPI [52].

The association between H. pylori and ulcer bleeding in people taking low-dose aspirin raises the question as to whether the main effect of the aspirin is to enhance bleeding from the ulcer caused by H pylori and would therefore the eradication of the H. pylori reduce or eliminate upper GI bleeding on aspirin? HEAT is an on-going study designed to test whether H. pylori eradication will reduce the incidence of bleeding peptic ulcers in patients taking aspirin [53]. HEAT is an outcomes study and uses cost saving innovations in the way the data and resources are utilised in order to save money and make it affordable. The trial is expected to complete in 2020.

Professor Hawkey concluded that inducing ulcer bleeding is the most common side effect from aspirin use and reformulating the aspirin does not work to prevent this. PPIs are effective but a H2 receptor antagonist a reasonable option if a PPI cannot be used. Aspirin is anti-haemastatic rather than ulcerogenic.

## Conclusion

Professor Patrono (Catholic University School of Medicine, Italy and University of Pennsylvania, USA) thanked everyone involved for a fantastic and very stimulating day of science and Bayer for supporting it. The presentations have illuminated aspects of the past history of aspirin but also most importantly its exciting potential in the future.

‘As I take over the chair of the Scientific Advisory Board of the International Aspirin Foundation from Professor Rothwell, I look forward to the Foundation representing an instrument for a joint venture of the medical/scientific community and the pharmaceutical industry to promote further research on aspirin and its multifaceted actions’.

## Conflicts of interest

Jaqui Walker was funded by the International Aspirin Foundation to attend this event in Berlin and received funding for the time required to write the meeting report.
